# Two clip-domain serine protease homologs, cSPH35 and cSPH242, act as a cofactor for prophenoloxidase-1 activation in *Drosophila melanogaster*

**DOI:** 10.3389/fimmu.2023.1244792

**Published:** 2023-09-15

**Authors:** Qiao Jin, Yang Wang, Haodong Yin, Haobo Jiang

**Affiliations:** Department of Entomology and Plant Pathology, Oklahoma State University, Stillwater, OK, United States

**Keywords:** clip domain, insect immunity, melanization, protease cascade, hemolymph protein

## Abstract

Insect phenoloxidases (POs) catalyze phenol oxygenation and *o*-diphenol oxidation to form reactive intermediates that kill invading pathogens and form melanin polymers. To reduce their toxicity to host cells, POs are produced as prophenoloxidases (PPOs) and activated by a serine protease cascade as required. In most insects studied so far, PPO activating proteases (PAPs) generate active POs in the presence of a high *M*_r_ cofactor, comprising two serine protease homologs (SPHs) each with a Gly residue replacing the catalytic Ser of an S1A serine protease (SP). These SPHs have a regulatory clip domain at the N-terminus, like most of the SP cascade members including PAPs. In *Drosophila*, PPO activation and PO-catalyzed melanization have been examined in genetic analyses but it is unclear if a cofactor is required for PPO activation. In this study, we produced the recombinant cSPH35 and cSPH242 precursors, activated them with *Manduca sexta* PAP3, and confirmed their predicted role as a cofactor for *Drosophila* PPO1 activation by MP2 (*i.e*., Sp7). The cleavage sites and mechanisms for complex formation and cofactor function are highly similar to those reported in *M. sexta*. In the presence of high *M*_r_ complexes of the cSPHs, PO at a high specific activity of 260 U/μg was generated *in vitro*. To complement the *in vitro* analysis, we measured hemolymph PO activity levels in wild-type flies, cSPH35, and cSPH242 RNAi lines. Compared with the wild-type flies, only 4.4% and 18% of the control PO level (26 U/μl) was detected in the cSPH35 and cSPH242 knockdowns, respectively. Consistently, percentages of adults with a melanin spot at the site of septic pricking were 82% in wild-type, 30% in cSPH35 RNAi, and 53% in cSPH242 RNAi lines; the survival rate of the control (45%) was significantly higher than those (30% and 15%) of the two RNAi lines. These data suggest that *Drosophila* cSPH35 and cSPH242 are components of a cofactor for MP2-mediated PPO1 activation, which are indispensable for early melanization in adults.

## 1 Introduction

Proteolytic activation of prophenoloxidase (PPO) and PO-catalyzed pathogen killing and melanin formation have been investigated for more than fifty years in various insects, such as lepidopterans, coleopterans, and dipterans (1–5). Some progress has been made regarding the mechanisms of the activation reaction. In the silkworm *Bombyx mori*, PPO activating enzyme (a cSP or clip-domain serine protease) alone generated highly active PO (6). *Manduca sexta* PPO activating protease-1, 2, or 3 (PAP1/2/3) activated the PPOs in the presence of a cofactor containing clip-domain serine protease homologs (cSPHs) (7–11). The high *M*_r_ cofactor is composed of a type I SPH (*i.e*., 1a, 1b, 4, or 101) and a type II SPH (*i.e*., 2) (12). In another lepidopteran species, *Helicoverpa armigera* cSPH11 (SPHI) and cSPH50 (SPHII) largely enhanced PPO activation by cSP6 (13). In the Order of Coleoptera, *Holotrichia diomphalia* PPO activating factor-1 (cSP) and -2 (cSPH) together activated the PPOs (14, 15), and so did *Tenebrio molitor* Spätzle processing enzyme and serine protease homolog-1 (16, 17). In the mosquito *Aedes aegypti*, CLIPB9 activated PPO3 along with CLIPA14 (SPHI) to a level higher than CLIPB9 alone did (18). While PPO activation by PAP requires a cofactor in most insects studied, it is unknown if a complex of SPHI and SPHII could lead to a maximal level of PO in *B. mori*, *H. diomphalia*, *T. molitor*, and *A. aegypti*. Neither is it clear how knocking-down one of the cSPHs by RNAi may affect melanization and survival of pathogenic bacteria *in vivo*. Beginning to fill this knowledge gap, we extended *in vitro* studies of the cofactor-assisted PPO activation to *Drosophila melanogaster*.

*D. melanogaster* has three PPO genes in the genome, coding for three proenzymes with distinct functions during wound healing, microbial infection, and melanotic encapsulation of parasitoid wasps (19, 20). PPO1 and PPO2 are produced in crystal cells, and PPO3 in lamellocytes may not need a protease for spontaneous activation (21). While PPO1 is made for rapid, early delivery of PO activity upon injury, PPO2 is stored in a crystalline form for deployment in the later phase of infection-induced melanization. PPO3 participates in the encapsulation of wasp eggs, in association with PPO2. Genetic analyses showed that MP1, MP2 (also known as Sp7 or PAE1), and Hayan lead to PPO1 activation (22–26). Defects in these genes or failure to make enough of the proteins differentially affected the survival of the flies after infection with bacteria and fungi. Subsequent experiments using single, double, and triple deletion mutants provided compelling evidence that PPO activation is indispensable for the *Drosophila* defense response against bacteria, fungi, and parasitoids (19, 20). Furthermore, the authors pointed out that a significant fraction of the Toll-induced killing of bacteria in adults is likely caused by PO activity, which is consistent with the *in vitro* killing effects (27, 28).

The approach combining the biochemical study of recombinant proteins can be useful for solving problems that are hard to disentangle by genetic analysis alone. For instance, bovine coagulation factor Xa activated the purified proMP2x_a_ expressed in *Drosophila* S2 cells (29). The active MP2x_a_ cleaved PPO1 from *E. coli* likely at the classical activation site between Arg^52^ and Phe^53^ (30), suggesting that MP2 is a PAP of the PPO1. Despite the fact that half of the correctly folded PPO1 was processed, no PO activity was reported in that paper (29). Based on the cofactor studies in other insects and the low PO activity generated by *Drosophila* MP2x_a_ alone, we hypothesized that one or two SPHs may act as a cofactor for PPO activation in *Drosophila*. Therefore, we expressed in insect cells the precursors of cSPH35 and cSPH242, the only SPHI and SPHII orthologs in *D. melanogaster* (31), and then activated them using *M. sexta* PAP3 (32). Using MP2x_a_ and PPO1 in the presence of these two cSPHs, we explored the mechanism of the PPO activation and demonstrated the cofactor’s existence and functional importance in *Drosophila*. To test the roles of these two SPHs *in vivo*, we knocked down their expression in their RNAi lines and observed the phenotype of significant decreases in PO activity, melanin formation, and survival rate of the flies infected with a strain of *Enterococcus faecalis*.

## 2 Materials and methods

### 2.1 Acquisition, maintenance, and crossing of fly lines for RNAi knockdown of cSPH35 and cSPH242 expression

Wild-type (Oregon-R) and transgenic strains of *D. melanogaster* were purchased from Carolina Biological and Bloomington Drosophila Stock Center (https://bdsc.indiana.edu/), respectively. BL4414 expresses GAL4 ubiquitously under the control of the Act5C promoter. BL52875 and BL44268 express dsRNA for RNAi of cSPH35 (CG5390) and cSPH242 (CG40160) under the control of GAL4, which binds UAS enhancer (33). The flies were maintained on a standard diet at 25°C (34). Newly emerged male adults (BL4414) with the GAL4 driver were mated with virgin female adults (BL52875 or BL44268) and F1 offspring from these crosses with straight wings were collected 1–2 day-post-eclosion and transferred onto the fresh diet.

### 2.2 Determination of cSPH35 and cSPH242 mRNA levels by qRT-PCR analysis

For knockdown verification, total RNA samples were extracted from the control (BL4114) and F1 adults (*i.e*., cSPH35kd and cSPH242kd) five days after emergence. cDNA samples, each equivalent to starting with 50 ng total RNA, were incubated with 1× iTag Universal SYBR Green Supermix (Bio-Rad) and specific primers (0.5 mM each) in triplicate in each reaction (10 μl). The primers were: J1091 (5’-GTGGTCCATTCCACTTCCGT) and J1092 (5’-TCATCATGTCGCGTGGATCA) for ribosomal protein L32 (rpL32) as a reference; J1971 (5’-GAAAGTCCATTCACCCTCCA) and J1972 (5’-TCCTTGCCGAACTTGTTCTT) for cSPH35; J1973 (5’-AAGACGTTGAATCCGACACC) and J1974 (5’-CCGCTTCATTTTGCGATACT) for cSPH242. Each primer pair was tested to ensure amplification of the target gene only. Amplification efficiencies were determined individually and confirmed to be 90−110%. Thermal cycling conditions were 95°C for 2 min and 40 cycles of 95°C 10 s and 60°C 30 s. After PCR was completed on a CFX Connect Real-Time PCR Detection System (Bio-Rad), melting curves of the products in all reactions were examined to ensure proper shape and Tm values. The mRNA levels were first normalized against the internal control of rpL32 using corresponding Ct values for the same cDNA samples and the relative mRNA levels were calculated as 2^-ΔCt^, where ΔCt = Ct _cSPH35/242_ - Ct _rpL32_. The lowest level of cSPH35 or cSPH242 mRNA in a dataset was then used as a reference (set at 1.00) to calculate mRNA fold differences (*i.e*., 2^-ΔΔCt^) for target genes in all the samples.

The same method was used to measure relative fold changes of the cSPH35 and cSPH242 transcript levels in wild-type thrid instar larvae, day 2 pupae, and day 2−3 adults for comparison. Similarly, immune inducibility was examined using naïve and infected wild-type adults. At 24 h before total RNA extraction, day 4–5 adult flies in the infection group were pricked with a 0.1 mm stainless needle in the thorax with live *Micrococcus luteus* (A_600 _= 100).

### 2.3 cDNA cloning and baculovirus construction for producing cSPH35, cSPH242, and MP2x_a_ precursors

A cSPH242 cDNA fragment was amplified from FBpp0112536 (https://flybase.org/) using primers J1681 (5’-TCATATGGCTCCTCAGCAGAAC) and J1682 (5’-ACTCGAGTGCGGTGTACACAG). The product was cloned into pGEM-T vector (Promega) and, after sequence validation, the *Nde* I-*Xho* I fragment was subcloned into the same sites in pMFFMH6 (Wang et al., unpublished data), a derivative of pMFH6 (35) with FLAG (DYKDDDDK) and c-myc (EQKLISEEDL) tags. cSPH242/pMFFMH6 was used to generate a baculovirus to express pro-cSPH242 (GIHDYKDDDDKHMAPQQN … AVYTALEQKLISEEDLHHHHHH, where the underlined part is encoded by the cDNA, 429 residues, 47,072 Da, pI: 5.52) in insect cells (36). A cSPH35 cDNA fragment was amplified from plasmid FBpp0079653 using J1685 (5’-CATATGCAGGACTCTTCCTTG) and J1686 (5’-TTGCTCGAGGGGTGTATAGTGCCT). After TA cloning and sequence confirmation, the *Nde* I-*Xho* I fragment was subcloned into the same sites in pMFFMH6 to generate a baculovirus for producing pro-cSPH35 (GIHDYKDDDDKHMQDSSL … RHYTPLEQKLISEEDLHHHHHH, 418 residues, 46,600 Da, pI: 5.40) in Sf9 cells. An MP2 cDNA fragment was amplified from MP2x_a_/pFastBac1 (29) using J1551 (5’-CATATGCAAGGAAGTTGTAGG) and J1552 (5’-CTCGAGGGGACGAATGGTCTC). After TA cloning and sequence validation, the *Nde* I-*Xho* I region was inserted into pMFFMH6 to generate plasmid, bacmid, and then baculovirus for producing proMP2x_a_ (GIHDYKDDDDKHMQGSCR … ETIRPLEQKLISEEDLHHHHHH, IEGR replacing FSNK, 394 residues, 43,895 kDa, pI: 5.57) in insect cells.

### 2.4 Expression and purification of the recombinant cSPH35, cSPH242, and MP2x_a_ precursors

Sf9 cells at 2.4×10^6^ cells/ml in 300 ml of insect serum-free medium (Thermo Fisher) were separately infected with the baculovirus stocks at a multiplicity of infection of 10 and grown at 27°C for 96 h with gentle agitation at 100 rpm. After the cells were pelleted by centrifugation at 5,000×*g* for 10 min, the pH of the conditioned media was adjusted to pH 6.4 with HCl. Cell debris and fine particles were spun down by centrifugation at 22,100×*g* for 20 min and the supernatant was mixed with an equal volume of 1 mM imidazole in water. The diluted solution was applied to a dextran sulfate-Sepharose column (20 ml bed volume) at a flow rate of 1.5 ml/min. Following a washing step with 100 ml of 0.01% Tween-20, 1 mM benzamidine in 10 mM potassium phosphate, pH 6.4, bound proteins were eluted with a linear gradient of 0−1.0 M NaCl in 80 ml of the same buffer. After SDS-PAGE and immunoblot analysis, the pro-cSPH fractions were combined, adjusted to pH 7.5, loaded onto a 2 ml Ni^2+^-nitrilotriacetic acid agarose column, and washed with 15 ml of 50 mM sodium phosphate, pH 7.5. Bound proteins were eluted with a gradient of 0−0.3 M imidazole in 20 ml of the same buffer. The pro-cSPH fractions were pooled, dialyzed against 20 mM Tris-HCl, pH 7.5, and concentrated on Amicon Ultra-30 centrifugal filter devices (Millipore). Aliquots of the proteins were flash-frozen in liquid nitrogen prior to long-term storage at -80°C. The MP2x_a_ precursor was produced in Sf9 cells infected by the recombinant virus and purified from the culture medium by following the same procedure.

### 2.5 Cleavage activation of cSPH242 and cSPH35 precursors by *M. sexta* PAP3

*M. sexta* PAP3 was isolated from pharate pupal hemolymph (10) and used to activate recombinant proPAP3 (32). Freshly prepared PAP3 was incubated with the purified *D. melanogaster* pro-cSPH242 and pro-cSPH35. The cleavage reactions, controls, and *M*_r_ markers were separated by 10% SDS and native PAGE, followed by electrotransfer and immunoblotting using antibodies against the hexahistidine tag. To better understand the process of PAP3 cleavage and high *M*_r_ SPH complex formation, aliquots of the pro-cSPHs were incubated with different amounts of PAP3 for 1 h at 37°C. The mixtures and PAP3 control were resolved by 10% SDS and native PAGE, transferred onto nitrocellulose membranes, and detected using diluted (His)_6_ antiserum as primary antibody, goat-anti-mouse IgG conjugated to alkaline phosphatase (Bio-Rad) as secondary antibody, and a BCIP-NBT substrate kit (Bio-Rad) for color development. Association states of the precursors and cleaved forms of cSPH35 and cSPH242 were determined by one-dimensional electrophoresis on a series of polyacrylamide gels under non-denaturing conditions (37).

### 2.6 Identification of the PAP3 cleavage sites in cSPH242 and cSPH35 by LC-MS/MS analysis

The purified pro-cSPHs (*ca*. 10 μg) were incubated with PAP3 (1.0 μg) for 1 h at 37 °C. The mixtures and negative controls of the pro-cSPHs were denatured in urea and digested with chymotrypsin or V8 protease overnight at 37 °C (38). The resulting peptides were desalted using Omix C18 affinity media, dried, and dissolved in mobile phase A (0.1% formic acid in H_2_O). The samples were loaded onto an Acclaim PepMap RSLC C18 column (75 μm × 50 cm, Thermo Fisher) for data-dependent LC-MS/MS analysis as described previously (39). Peptides in each sample were separated via a gradient of 0–35% mobile phase B (0.1% formic acid in 80% AcCN) developed over 120 min (12). The survey scans were followed by both HCD and CID collisional MS/MS events triggered from the hypothetical peptide ions, with scanning of collisional fragments at 15,000 resolutions in the Orbitrap sector.

For identifying the PAP3 cleavage sites in cSPH242 and cSPH35, a database combining *D. melanogaster* pro-cSPH242 and pro-cSPH35 and background proteins from *M. sexta*, human, and insect cells were constructed, and peptide spectrum matches were reviewed in Byonic to detect peptides not cut by nonspecific proteases. The details of peak area calculation were described previously to quantify characteristic peptides released by PAP3 and chymotrypsin/V8 protease (12). The original MS imaging data were deposited at a public repository (https://doi.org/10.6084/m9.figshare.23786949).

### 2.7 Activation of proMP2x_a_ and proPAP3 followed by an amidase assay

The purified poMP2x_a_ (0.44 µg) was incubated with Factor Xa (0.1 µg) at 37 °C for 1 h in 10 μl buffer A. Half of the reaction mixture and controls (proMP2x_a_ and Factor Xa only) were separately incubated with 150 μl 25 µM acetyl-Ile-Glu-Ala-Arg-*p*-nitroanilide (IEAR*p*NA, Sigma-Aldrich, A0180) in buffer A. Absorbance of the mixtures was monitored at 405 nm on a microplate reader. One unit of the hydrolytic activity is defined as the amount of enzyme causing ΔA_405_/min of 0.001. To obtain active PAP3, proPAP3 (0.4 µg/µl, 1 µl) was incubated with active PAP3 (40 ng/µl, 1 µl) in 10 μl buffer A at 37°C for 1 h, half of the mixture (0.2 µg) was used to test IEARase activity, as described above for MP2x_a_.

### 2.8 Drosophila PPO1 expression, activation, and PO activity assay

*D. melanogaster* PPO1 was expressed in *E. coli* BL21 harboring PPO1/pET28a and purified by affinity chromatography on a Ni-NTA agarose column (30). The active MP2xa was produced by preincubating proMP2x_a_ (440 ng/μl, 1 μl) with Factor Xa (100 ng/μl, 1 μl) at 37°C for 1h. The two cSPHs were generated by treating pro-cSPH35 and pro-cSPH242 (each at 200 ng/μl, 1 μl) with *M. sexta* PAP3 (10 ng/μl, 1 μl) at 25°C for 1h. *M. sexta* serpin-3 (100 ng/μl, 1 μl) was added to the processed cSPH35 and cSPH242 and incubated at 25°C for 30 min to block PAP3 activity. To activate *Drosophila* PPO1 under different conditions, PPO1 (1 μl, 270 μg/ml, from *E. coli*) was processed by pretreated MP2x_a_ (200 ng/μl, 1 μl) with or without cleaved cSPH35 and cSPH242 on ice for 1 h in a total volume of 25 μl buffer A (0.001% Tween-20, pH 7.5, 20 mM Tris-HCl, 5 mM CaCl_2_). PO activity was determined on a microplate reader immediately after 150 μl of 2.0 mM dopamine in 50 mM sodium phosphate, pH 6.5, was added to each sample well (8). Immunoblot analysis was performed using the antibody against *Drosophila* PO1 fused with glutathione S-transferase (26), which did not crossreact with PO2 or PO3.

### 2.9 Hemolymph preparation for DmPPO1 immunoblot analysis, melanization observation, and PO activity measurement

A hole was pierced with a 25-gauge needle in the bottom of a 0.5 ml microfuge tube before placing it in a 1.5 ml tube with its lid removed. Twenty adults were pricked in the thorax with a fine tungsten needle, placed in the small tube, and centrifuged at 5000 rpm for 1 min at 4°C. After discarding the 0.5 ml tube, 1.0 µl of hemolymph in the large tube was transferred to 10 µl buffer A containing 0.1% 1-phenyl-2-thiourea (PTU). After 30 min at room temperature, the mixtures from BL4414 and the two RNAi lines were subjected to 7.5% SDS-PAGE and immunoblot analysis using 1:2000 diluted *D. melanogaster* PPO1 antiserum (30), a gift from Dr. Erjun Ling at the Shanghai Institute of Plant Physiology and Ecology. Hemolymph samples (1.0 µl, without PTU) from the three groups of adults were also transferred to wells of a microplate for PO activity measurement. After 150 μl of 2.0 mM dopamine solution was added (*Section 2.8*), PO activities were assayed immediately on a plate reader as described before (8). Spontaneous melanization of the hemolymph was observed at 30 min after hemolymph samples (5 µl each, without PTU) were collected from 50 adults in the three groups, transferred into microplate wells, and incubated at room temperature before imaging.

### 2.10 Infection of cSPH35 and cSP242 RNAi flies with *E. faecalis*

An *E. faecalis* glycerol stock, a gift from Dr. Zhen Zou at the Institute of Zoology, Chinese Academy of Sciences, was revived on an LB agar plate. A single colony was inoculated into 3 mL of LB liquid medium and grown overnight at 37°C with shaking. The bacteria were spun down for 1 min at 15,000×*g* and the pellet was resuspended in fresh LB to a final OD_600_ of 1.2 or 0.8 for different tests (40). In the survival test, 4–5-day-old adults in the control (BL4414) and RNAi lines were pricked in the thorax with a needle (0.1-mm stainless steel needle) tinted with *E. faecalis* at OD_600_ = 1.2 (41). The number of survivors in each group was counted daily from day 1 to day 10. In the melanization test, an *E. faecalis* suspension (OD_600_ = 0.8) was used to infect adults in the three groups under the same conditions. Bacteria at the lower dosage induced melanization but did not kill the flies. Black pigment deposited at the wound site was observed 2 days after infection under an Olympus BH2-RFCA microscope and pictures were taken using a 9MP microscope digital camera (Amscope). Numbers of flies with and without a melanin spot were recorded for three batches of flies in BL4414 and F1 offspring of the two crossed lines (*Section 2.1*). Besides, the microscope and camera were used to detect and record cuticle decoloring in some F1 offspring of the crossed line with cSPH242 knockdown (*Section 2.1*). For comparison, a series of images of the 4–5-day-old adult flies in the BL4414 and cSPH35 RNAi lines were also captured and merged into one using ImageJ (https://imagej.nih.gov/ij/).

## 3 Results

### 3.1 Rationale for studying *D. melanogaster* cSPH35 and cSPH242

Among the eighteen clip-domain SPH genes in *Drosophila*, cSPH35 and cSPH242 are 1:4 and 1:1 orthologous to *M. sexta* SPH1a, 1b, 4, and 101 (SPHIs) and SPH2 (SPHII) (12, 31), respectively. A new phylogenetic analysis indicated that, except for *D. suzukii* SPHIa−c and *D. elegans* SPHIIa, b, all fifteen *Drosophila* species have a pair of SPHI and SPHII genes in their genomes (**Figure S1**). In the superfamily Tephritoidea, the olive and Mediterranean fruit flies also possess a single pair of SPHI and SPHII genes. The house fly *M. domestica* has three SPHs (Ia, Ib, and II). In comparison, expansions of the orthologous genes in mosquitoes and lepidopteran insects are remarkable (12, 42).

**Figure 1:**
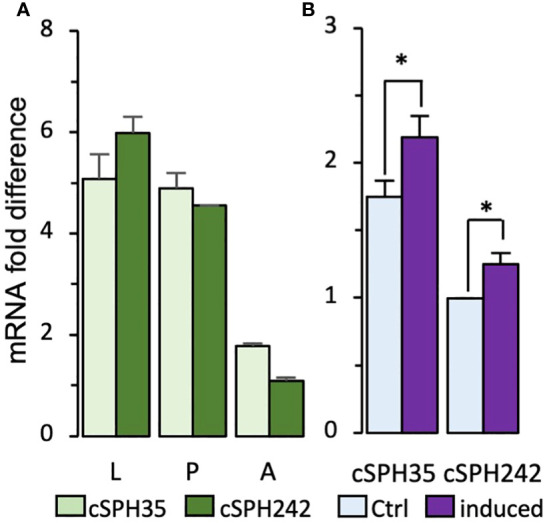
Quantitative RT-PCR analysis of *D melanogastor* cSPH35 and cSPH242 transcript levels. **(A)** The mRNA levels in 3^rd^ instar larvae (L), day 2 pupae (P), and day 2–3 adults **(A)** were determined by qRT-PCR, calculated using ΔΔCt values of cSPH35, cSPH242, and rpL32 (control) for each of the three biological replicates, and plotted in the bar graph, as mean ± SEM (n = 3) (*Section 2.2*). **(B)** Immune inducibility. Total RNA samples from naive and immune-challenged adults were analyzed similarly to measure cSPH mRNA fold changes. Statistical significance of the pairwise comparisons was analyzed using Student’s t-test (*, *p* < 0.05).

Based on the mass spectrometric data on *M. sexta* PPOs, PAPs, and SPHI-II (12, 43), we indicated that *Drosophila* cSPH35 and cSPH242 proteins may exist at high levels to act as components of the putative cofactor. Examination of the mRNA profiles (**Figure S2A**) revealed that, except for the early embryonic stage (0–10 h), their expression patterns were highly similar throughout the life span. We further confirmed by qRT-PCR analysis that levels of the two cSPH transcripts are comparable with each other in larvae, pupae, and adults (**Figure 1A**). With ΔCt values ranging from 4 to 7, the cSPH mRNA levels are high, in comparison to the vastly abundant rpL32. These data are consistent with their high protein abundances at the whole-body level in embryonic, larval, pupal, and adult stages (**Figure S2B**) (31, 44). In contrast to the low expression of cSPH35 in early embryos (0–10 h, **Figure S1A**), moderate cSPH242 transcript and protein levels in the same stage were observed. As shown in **Figure 1B**, there is an association of *Drosophila* cSPH35 and cSPH242 with innate immunity, since both cSPH35 and cSPH242 mRNA levels increased significantly after exposure to live *M. luteus*.

### 3.2 Recombinant expression and characterization of the pro-cSPHs and proMP2x_a_

**Figure 2:**
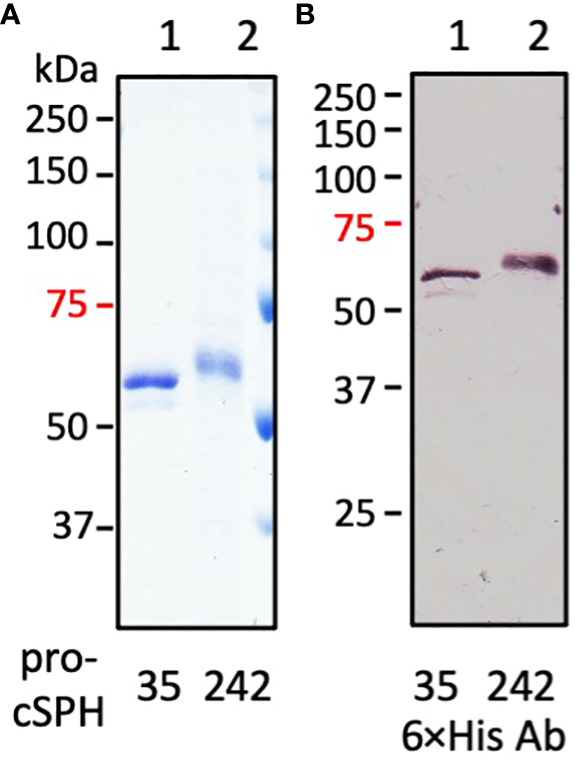
SDS-PAGE and immunoblot analyses of the purified precursors of *D. melanogastor* cSPH35 and cSPH242. The recombinant pro-cSPHs were resolved by 10% SDS-PAGE under the reducing condition followed with Coomassie brilliant blue staining (panel **A**, 1 μg per lane) or immunoblotting (panel **B**, 200 ng per lane) using 1:1000 diluted antiserum against hexahistidine affinity tag. Lane 1: pro-cSPH35; lane 2: pro-cSPH242; Positions and sizes (in kDa) of the prestained *M*_r_ standards are marked on the *left*, with the 75 kDa marker highlighted *red*.

To study the functions of cSPH35 and cSPH242 *in vitro*, we constructed two baculoviruses to infect Sf9 cells for protein production and purification from the cultures. Pro-cSPH35 (0.8 mg) and pro-cSPH242 (1.0 mg) were isolated from the conditioned media (100 ml each), which migrated to the 60 and 63 kDa positions on a 10% SDS polyacrylamide gel (**Figure 2**). Major deviations from their calculated *M*_r_’s (46.6 and 47.1 kDa) suggested they were modified post-translationally, by glycosylation likely. As shown in native PAGE, most of the precursors existed as a monomer (**Table 1**) and some pro-cSPH242 were in higher association states (**Figure S7**). We also expressed and purified proMP2x_a_ (0.5 mg) from the culture medium (150 ml). The protein migrated as a single band at 50 kDa on 10% SDS-PAGE (**Figure S3A**), larger than its calculated *M*_r_ of 43.9 kDa.

**Table 1:** Sizes of the native cSPH35 and cSPH242 after cleavage by *M. sexta* PAP3 ^‡^.

sample	*M*_r_ of the proteins or their complexes (kDa)
pro-cSPH35	57
pro-cSPH35 + PAP3	**120, 200, 260**
pro-cSPH242	60
pro-cSPH242 + PAP3	55, **150, 260, 380**
pro-cSPH35 + pro-cSPH242 + PAP3	**140, 360, 440, 530**

^‡^ Molecular masses (M_r_) of the proSPHs and their cleavage products were determined by native PAGE on 4.5%, 7%, 10%, 12%, and 15% discontinuous gels. Protein standards (29 kDa carbon anhydrase, 66 kDa bovine serum albumin, 200 kDa β-amylase, 443 kDa apoferintin, and 669 kDa thyroglobulin) were run alongside in individual lanes. Sizes of the precursors and complexes of their cleavage products were deduced from their relative mobilities as described in the legend to **Figure S7**, with estimated M_r_’s of the complexes of cleaved cSPH242, cSPH35, and both in bold font.

### 3.3 Proteolytic processing of the two pro-cSPHs by *M. sexta* PAP3

*Drosophila* MP2 acts as a PAP to directly activate PPO1 (29) and some insect PAPs also generate their own cofactors by cleaving SPHI and SPHII precursors (17, 32, 35). Therefore, we activated proMP2x_a_ by bovine factor X_a_ and detected its catalytic domain at 34 kDa and an increase in IEAR*p*NA hydrolysis from 1.33 U to 2.63 U (**Figure S3**). However the active MP2x_a_ failed to cleave pro-cSPH35 or pro-cSPH242 and did not cause a remarkable association of the monomers (**Figure S4**).

**Figure 3:**
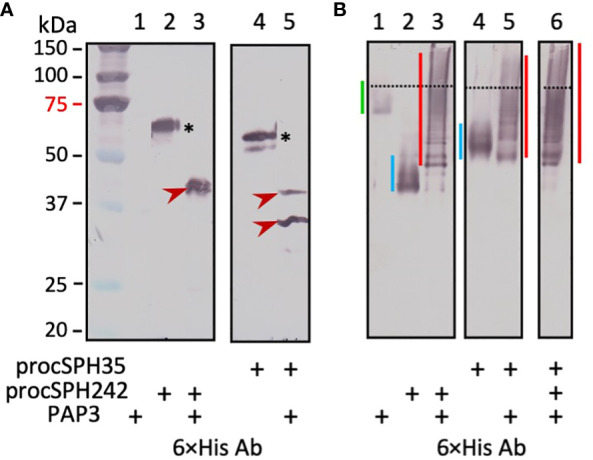
Immunoblot analysis of the PAP3 cleavage products from *D melanogastor* cSPH35 and cSPH242 precursors after 10% SDS **(A)** and native **(B)** PAGE. The pro-cSPHs (200 ng/μl, 1 μl) were separately incubated with *M. sexta* PAP3 (40 ng/μl, 1 μl) in 10 μl buffer A at 37°C for 1 (h) The reaction mixtures and controls (40 ng PAP3 and 200 ng proSPH alone) were treated with 1×SDS sample buffer at 95°C for 5 min or the same buffer lacking SDS and DTT at 25°C for 5 min prior to 10% SDS-PAGE and 10% native PAGE, respectively. After electrotransfer, immunoblot analysis was performed using 1:1000 diluted antiserum against hexahistidine tag. In panel **(A)**, pro-cSPH242 (lane 2), pro-cSPH35 (lane 4), and major cleavage products (lanes 3 and 5) are marked by *asterisks* and *arrowheads*, respectively. Positions and sizes of the prestained *M*_r_ standards are indicated, with the 75 kDa marker highlighted *red*. In panel **(B)**, the dashed line divides the stacking and separating gels. The smeared bands of PAP3, proSPHs, and cleavage products are marked by *green*, *blue*, and *red vertical bars*, respectively.

We then tested whether *Manduca* PAP3, an activator of the proSPHs (1a, 1b, 2, 4, and 101) (12), could activate *Drosophila* pro-cSPH35 and pro-cSPH242. To our surprise, the processing of pro-cSPHs was complete: a 42 kDa fragment was generated in the reaction of pro-cSPH242 and PAP3 and recognized by the hexahistidine antibody, whereas two carboxyl-terminal fragments of cSPH35 were detected at 40 and 30 kDa positions by the same antibody (**Figure 3A**). Native PAGE and immunoblot analysis indicated that the processed proteins formed a smear of high *M*_r_ complexes extending from the stacking gel (**Figure 3B**).

**Figure 4:**
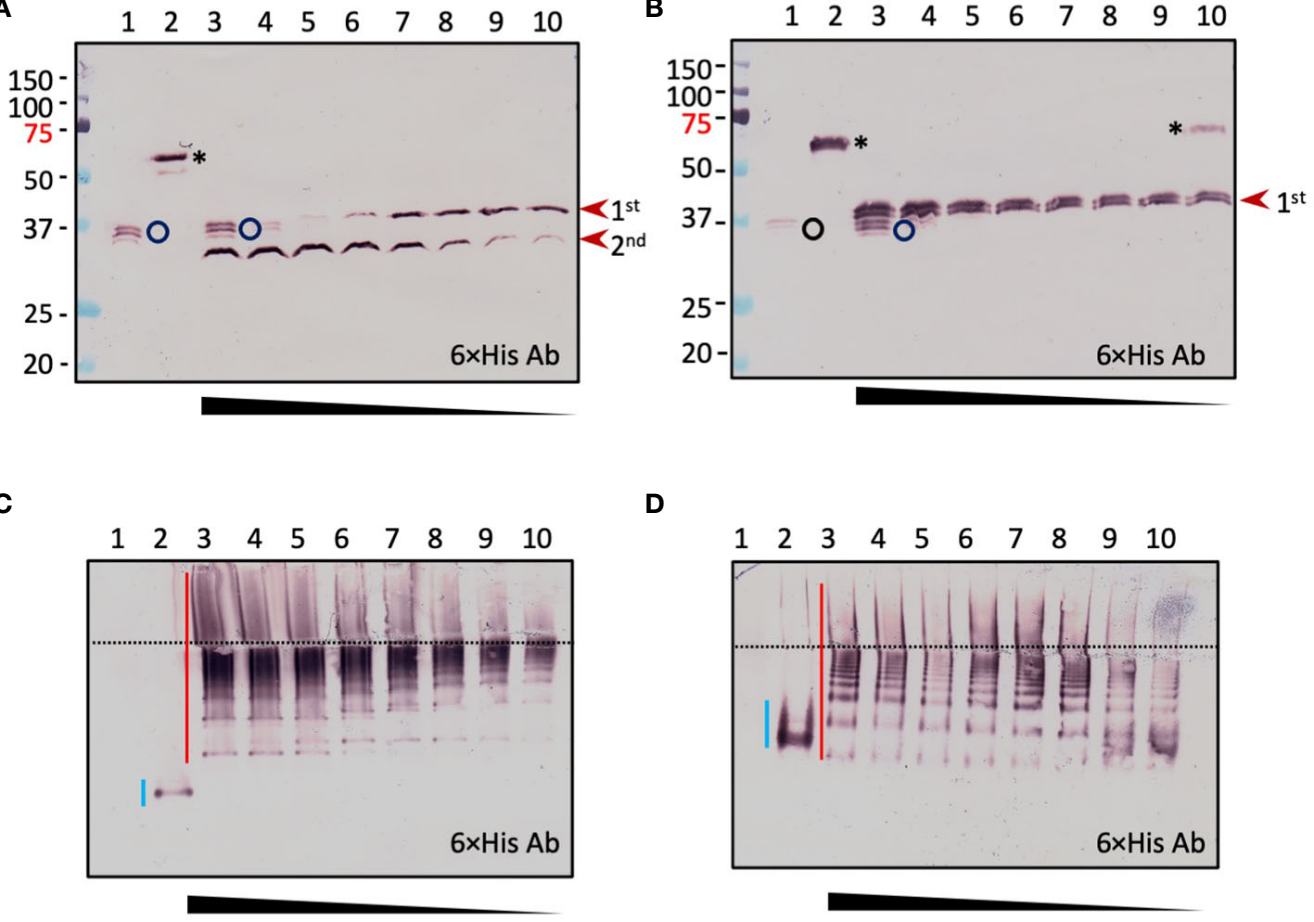
Concentration-dependent PAP3 processing of *D melanogaster* cSPH35 **(A, C)** and cSPH242 **(B, D)** precursors analyzed by 10% SDS and native PAGE followed by immunoblotting. Aliquots of the purified pro-cSPHs (200 ng) were incubated with 80 (lane 3), 40 (lane 4), 20 (lane 5), 10 (lane 6), 5 (lane 7), 2.5 (lane 8), 1.2 (lane 9), 0.6 (lane 10), and 0 (lane 2) ng of PAP3 in 10 μl buffer A at 37°C for 1 (h) The mixtures and PAP3 control (80 ng, lane 1) were subjected to 10% SDS-PAGE **(A, B)** and 10% native PAGE **(C, D)**, as described in the legend to **Figure 3**. After electrotransfer, immunoblot analysis was performed using antiserum against hexahistidine tag as primary antibody. In **(A, B)**, PAP3, proSPHs, and major products from the first and second cleavages are marked by *circle*, *asterisk*, and *arrowheads*, respectively. Positions and sizes of the prestained *M*_r_ standards are indicated, with the 75 kDa marker highlighted *red*. In **(C, D)**, the dashed line divides the stacking and separating gels. The smeared bands of pro-cSPHs, and cleavage products are marked by *blue* and *red vertical bars*, respectively.

To examine the dynamic processes of pro-cSPH cleavage, we separately incubated aliquots of the pro-cSPH35 or pro-cSPH242 with decreasing amounts of PAP3, resolved the reaction mixtures by 10% SDS-PAGE, and performed immunoblot analysis using the hexahistidine antibody (**Figure 4**). At the lowest PAP3 level, the 40 kDa cSPH35 band was major and the 30 kDa band was minor (panel A, lanes 9 and 10), suggesting that cleavage first occurred at site-1. As the PAP3 concentration increased, the intensity of the 30 kDa cSPH35 increased while the 40 kDa band decreased and disappeared due to cleavage at site-2 (panel A, lanes 3−8). On the native PAGE gel, cleavage of the pro-cSPH35 monomer (panel C, lane 2) at the two sites yielded a smear in the stacking gel and a ladder in the separating gel, and the ratios of smear and ladder increased as more PAP3 was added (**Figure 4C**). In comparison, pro-cSPH242 processing at a single site is much simpler: as PAP3 increased and pro-cSPH242 disappeared, the 42 kDa band reached the highest level (**Figure 4B**, lanes 3–10). On the native PAGE gel, there was a band migrating slightly faster than pro-cSPH242 (**Figure 4D**). As the monomer decreased in lanes 10 and 9, the ladder of cSPH242 complexes extended into the stacking gel in lanes 8, 7, 6, and so on. These results clearly indicated that PAP3 efficiently cleaved the pro-cSPHs and readily changed the association states of the cSPHs, orthologs of the *Manduca* SPHIs, and SPHII.

### 3.4 Identification of the cleavage sites in *Drosophila* cSPH35 and cSPH242

Similar to *Drosophila* cSPH35, *M. sexta* proSPH1b was cleaved by PAP3 first at R^82^ and then at R^133^ (9, 11, 12). Like cSPH242, *Manduca* proSPH2 was activated at a single site (R^77^). Therefore, we activated pro-cSPH35 and pro-cSPH242 with PAP3, cleaved cSPH35 with V8 protease and cSPH242 with chymotrypsin, and performed LC-MS/MS analysis. Since PAP3 cleaves after R or K, V8 cleaves after D or E, and chymotrypsin cleaves after F, Y, W, M, or L, identification of the peptides released from PAP3-V8 protease and PAP3-chymotrypsin digestion would allow us to deduce the PAP3 cleavage sites in the cSPHs. Indeed, we detected three such peptides and acquired their secondary ion mass spectra (**Figure S8**) but not in the control pro-cSPH samples treated with chymotrypsin or V8 protease only (**Table 2**). By mapping these peptides to the mature proteins, we found that pro-cSPH35 was cleaved at R^81^ and K^128^ whereas pro-cSPH242 was cleaved at R^88^. These sites corresponded well with the activation sites in their orthologs from *M. sexta*.

**Table 2:** Determination of PAP3 cleavage sites in *D. melanogastor* cSPH35 and cSPH242 precursors.

sample	predicted cleavage site-1	peak area	predicted cleavage site-2	peak area
cSPH35+V8	IDIR^81^*LGTDAECKNYLD(L)	*n.d*.	VGFK^128^*ITGAVNQEAE(F)	*n.d.*
cSPH35+PAP3+V8	IDIR^81^* **LGTDAECKNYLD** (L)	4.61×10^9^	VGFK^128^* **ITGAVNQEAE** (F)	2.17×10^10^
pro-cSPH242+chy	(F)TEDGSFDGFGVIDIR^88^*FND	*n.d*.	none	*n.d*.
pro-cSPH242+PAP3+chy	(F) **TEDGSFDGFGVIDIR**^88^*FND	5.3×10^8^	none	*n.d.*

The PAP3-treated cSPH precursors were separately reacted with chymotrypsin (chy, cleavage after F or Y) and V8 protease (cleavage after D or E) to release the three peptides (bold and underlined) as identified by LC-MS/MS analysis (**Figure S8**). In the control samples of the pro-cSPHs reacted with chy or V8 only, these peptides were undetected (n.d.) at the same retention times. Peak areas of the three peptides are indicated.

### 3.5 PAP3-activated cSPH242 and cSPH35 act as a cofactor for *Drosophila* PPO1 activation

**Figure 5:**
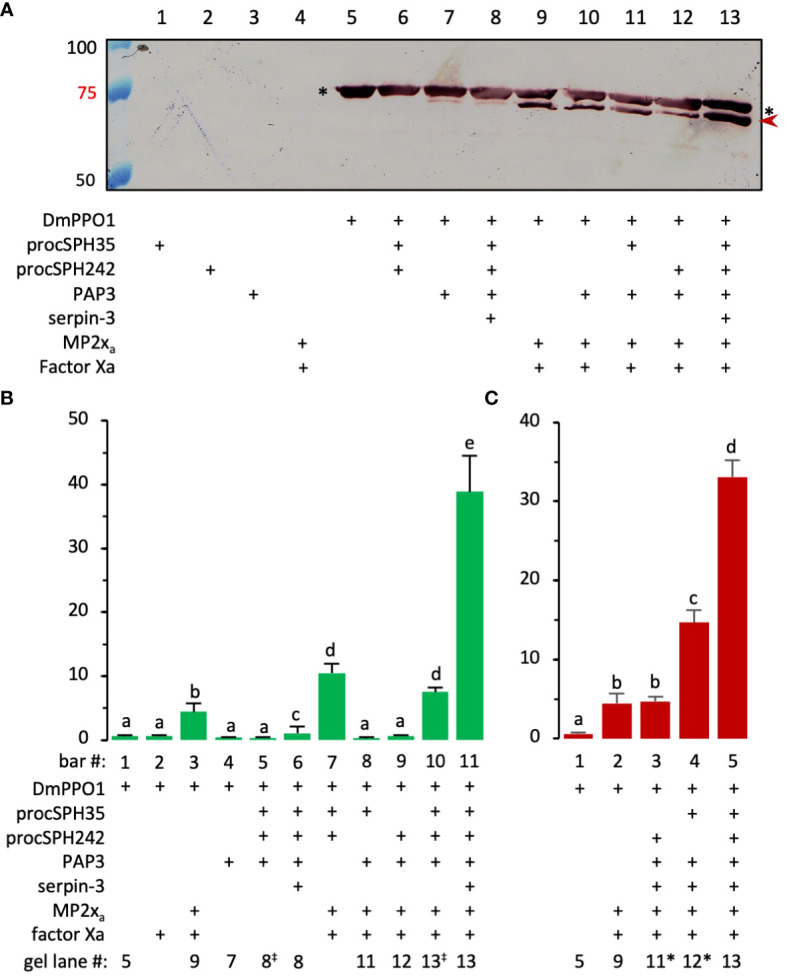
Effect of PAP3-treated pro-cSPH242 and pro-cSPH35 on *D melanogaster* PPO1 activation by MP2x_a_. **(A)** PPO cleavage by MP2x_a_. ProMP2x_a_ (0.44 μg) was activated by Factor Xa (0.1 μg) in buffer A (2 μl) for 1 h at 37°C. In another reaction, cSPH242 and cSPH35 precursors (400 ng each) were incubated with *M. sexta* PAP3 (20 ng) in buffer A (8 μl) at 25°C for 1 h, and PAP3 was then inhibited by *M. sexta* serpin-3 (100 ng/μl, 2 μl) at 25°C for 30 min. *D melanogastor* PPO1 (2 μl, 0.3 μg/μl) from *E coli* was incubated with active MP2x_a_ (400 ng) in the presence or absence of active cSPH242 and cSPH35 (400 ng each) on ice for 1 h in 10 μl buffer **(A)** Half of the mixture (lane 13) and controls (lanes 1−12) were subjected to 7% SDS-PAGE followed by immunoblotting using the specific PPO1 antibody (26). As indicated in panel A, the missing component(s) in the controls were replaced by the same volume of buffer **(A, B)** PPO1 activation. PO activities in some of the remaining samples were measured in 150 μl of 2.0 mM dopamine in 50 mM sodium phosphate, pH 6.5, using a microplate reader, and the activity data from three biological replicates (mean ± SEM, n = 3) were plotted in the bar graph. **(C)** Roles of the cSPHs. As described in panel B, a similar experiment was performed to assess the relative contributions of cSPH242 and cSPH35 in *D melanogaster* PPO1 activation. To facilitate comparison, corresponding lane numbers in panel **(A)** are indicated below the two bar graphs, with the slight changes indicated as “‡” or “*”. The statistical significance of the differences was determined by one-way ANOVA and indicated by different letters (a−e) for *p <*0.05.

*Drosophila* MP2x_a_ cleaved half of the recombinant PPO1 (0.38 μg) but no PO activity was reported (29). Our data showed that MP2x_a_ hardly processed the two pro-cSPHs (**Figure S4**). Can the PAP3-treated pro-cSPHs greatly enhance PPO1 activation by MP2x_a_? We found, although there was a clear increase in PPO1 cleavage by MP2x_a_ in the presence of both pro-cSPHs (**Figure S6**, lane 8 vs. lane 11), a small but significant enhancement in PPO activation (10.5 U) was observed (**Figure 5B**, bar 7). PAP3 slightly cleaved *Drosophila* PPO1 but did not increase PO activity (**Figure 5A**, lane 7; **Figure 5B**, bar 4). To obtain the active cofactor without interfering with the cleavage of DmPPO1 by MP2x_a_ (especially when the cSPHs were present), we blocked PAP3 with *M. sexta* serpin-3 (45), which did not inhibit MP2x_a_’s IEARase activity by forming an inactive complex (**Figure S5**). After serpin-3 inhibition, the PAP3-generated cSPHs were incubated with *Drosophila* PPO1 and MP2x_a_ (*i.e*., Factor Xa-treated proMP2x_a_). MP2x_a_ cleaved nearly half of the PPO1 and yielded a 38.9 U of PO at a specific activity of 260 U/μg (**Figure 5A**, lane 13; **Figure 5B**, bar 11), clearly demonstrating the cofactor role of the SPHI-II complexes versus their precursors (**Figure 5A**, lane 7). In comparison, PPO1 cleavage at 1/5, 1/5, and 1/10 levels (**Figure 5A**, lanes 9, 11, and 12) only yielded 4.4, 0.3, and 0.7 U of PO (**Figure 5B**, bars 3, 8, and 9), respectively. As we reported previously (46), there is no direct correlation between PPO cleavage extent and PO activity, and the presence of a high *M*_r_ cofactor of SPHI and SPHII is critically important for generating highly active PO.

To assess the relative importance of cSPH35 and cSPH242 to the cofactor activity, we activated one of the pro-cSPHs by PAP3, inhibited PAP3 using serpin-3, and then added PPO1 and MP2x_a_ to the mixture. As shown in **Figure 5C**, the removal of pro-cSPH242 and pro-cSPH35 caused an activity decrease from 33.1 U to 14.7 U and 4.7 U, respectively. Both cSPHs, cSPH35 in particular, are required for the cofactor function.

### 3.6 Effects of cSPH35 or cSPH242 knockdown on melanization and survival of infected adults

**Figure 6:**
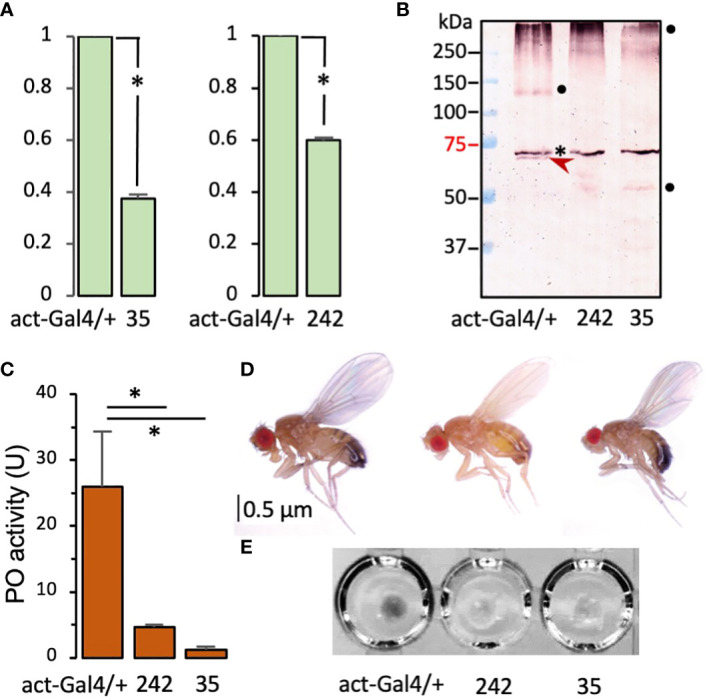
Effects of systemic knockdown of cSPH35 and cSPH242 expression in *D melanogastor* adults. **(A)** Decreases in the cSPH mRNA levels. The mRNA levels in adults (5 days after eclosion) were determined by qRT-PCR as described in *Section 2.2*. In relation to the control (set at 1.00), the cSPH transcript levels (mean ± SEM, n = 3) in the two RNAi lines are shown in the bar graphs. *, *p <*0.05 in Student’s t-test. **(B)** Reduction of PPO1 cleavage. Hemolymph samples from ten adults were separately collected from the control, cSPH35, and cSPH242 RNAi lines. The plasma samples (1.0 μl each) were incubated with 0.1% PTU in buffer A (10 μl) for 30 min at room temperature, separated by 7.5% SDS-PAGE, and detected by antibody to *D melanogaster* PPO1. PPO1 and its 73 kDa cleavage product are marked with *asterisk* and *arrowhead*, respectively. The high and low *M*_r_ bands (●) may represent cleaved PO in different association states. **(C)** PO activity decrease. Freshly collected hemolymph samples (1.0 μl from 10 individuals in the control and RNAi groups, three biological replicates) were assayed immediately for PO activity using dopamine as a substrate. The PO activities (mean ± SEM, n = 3) are shown in the bar graph, with * indicating *p <*0.05 in Student’s t-test. **(D)** Body color change. Images of adults from the control and RNAi lines, show that 30% of the cSPH242 knockdowns with low pigmentation. **(E)** Hemolymph melanization. Hemolymph samples (5 μl from 50 individuals) from the three groups of adults were transferred to wells of a microplate, where spontaneous melanization occurred in the control hemolymph within 5 min.

To examine phenotypes of the single knockdowns, we first measured the mRNA level changes in the two RNAi lines. Compared with the control, 40% and 60% of the cSPH35 and cSPH242 mRNA levels were detected in the adults (**Figure 6A**). After incubation at 25°C for 10 min with 0.1% PTU (a PO inhibitor that prevents protein crosslinking), nearly one fifth of the PPO1 in the control hemolymph was cleaved whereas no cleavage at the activation site of Arg^52^ was detected in the samples from cSPH242 or cSPH35 RNAi adults (**Figure 6B**). In another set of samples lacking PTU, PO activities were detected at 26.0, 4.7, and 1.2 U per μl of hemolymph from the control, cSPH242, and cSPH35 knockdown adults (**Figure 6C**). Consistently, hemolymph from wild-type flies turned black within 5 min at 25°C while samples from the RNAi lines stayed colorless for 60 min at least (**Figure 6E**). Some of the cSPH242 knockdowns appeared to have insufficient melanin deposition in the posterior abdominal segments (**Figure 6D**). This phenotype of light color is not found in the control or cSPH35 RNAi flies, suggesting that cSPH242 participates in cuticle pigmentation and its lower expression (**Figure 6A**) somehow led to insufficient melanin formation.

**Figure 7:**
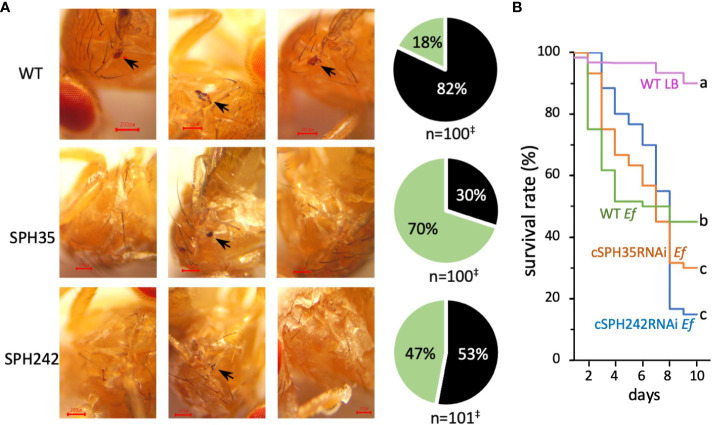
Effects of cSPH242 or cSPH35 knockdown on melanization and survival of infected *D melanogastor* adults. **(A)** Melanization at the site of septic pricking. Representative images of the control, cSPH35, and cSPH242 RNAi flies with or without a black spot (*black arrow*) in the wounded thorax at 24 h after exposure to live *E faecalis* (OD_600 =_ 0.8). Pie charts on the *right* show the percentages of flies in the three groups with (*black*) and without (*green*) a melanin spot. ^‡^, data pooled from three biological replicates; intergroup differences: *p <*0.0005 in Student’s t-test with Boniferoni correction. **(B)** Survival curves. Twenty-five flies from each group were pricked in the thorax with LB medium or live *E faecalis* (OD_600 =_ 1.2), transferred to clean vials with a fresh diet, and then incubated at 25°C. Survived flies were counted daily before transfer to new vials. The experiment was repeated at least three times and percentages of survived flies in each pooled group were shown with different time points (days 1–10 after treatment).

We studied the impacts of the cSPH knockdown on melanization at the site of septic pricking with *E. faecalis* (OD_600_ = 0.8). Eighty-two percent of the wild-type flies exhibited a black spot on the thorax two days after inoculation (**Figure 7A**). The percentage decreased to 53% in the RNAi line of cSPH242. Consistent with the lowest PO activity (**Figure 6C**), only 30% of the adult flies with cSPH35 expression knocked down had melanin deposited at the wound site. The intergroup differences were highly significant. To further investigate the role of these cSPHs in the immune response, we raised the dosage of *E. faecalis* (OD_600 =_ 1.2) and monitored the survival of infected adults for ten days. Compared with the wild-type flies, the survival rate decreased from 45% to 30% and 15% in the cSPH35 and cSPH242 RNAi lines (**Figure 7B**), respectively. The differences between the control and RNAi groups were statistically significant.

## 4 Discussion

### 4.1 Requirement of a cofactor for PPO activation in various insects

**Figure 8:**
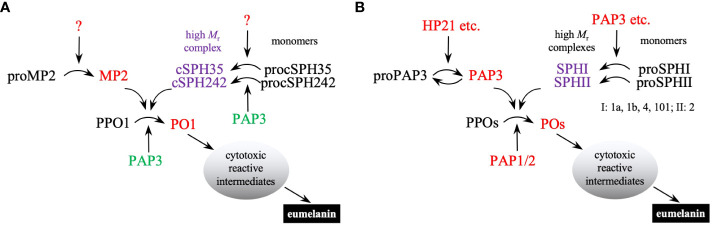
A conserved mechanism of PPO activation in *D melanogaster* **(A)** and *M. sexta* **(B)**. In *Drosophila*, unknown clip-domain SPs activate MP2, cSPH35, and cSPH242 precursors (*black* font). Active MP2 (*red* font for enzyme activity) and its cofactor (*purple* font for catalytic inactivity but cofactor activity) cleave PPO1 to generate highly active PO1. Under *in vitro* conditions, *M. sexta* PAP3 (*green* font) activates the *Drosophila* proSPH monomers to form high *M*_r_ complexes, which act as a cofactor for PPO1 activation by *M. sexta* PAP3. In *Manduca*, HP21 and other hemolymph proteases (including PAP3 itself) activate proPAP3. PAP3 and additional clip-domain SPs cleave proSPHIs and proSPHII to generate active cofactors for PPO activation by PAP1/2/3. Since multiple proteins perform the same function in each step, *M. sexta* PPO activation appears to be more complicated than in *Drosophila*. Once active POs are generated, they catalyze the formation of reactive intermediates to kill invading microbes and form melanin sheaths to sequester them.

Despite the apparent discrepancy in *B. mori* (6), it is now clear that PPO activation is mediated by a clip-domain SP (*i.e*., PAP) in the presence of one or two cSPHs in all insects studied so far. This seems to be a conserved mechanism that existed before the radiation of holometabolous insects, including beetles, moths, and mosquitoes (9, 12, 13, 15, 17, 18, 47). Guided by the orthologous relationships, we identified the two types of cSPHs in *D. melanogaster* (**Figure S1**) and demonstrated by *in vitro* and *in vivo* experiments that the mechanism is functional in the fruit fly as well (**Figure 8**). In most other *Drosophila* species, single orthologs of cSPH35 and cSPH242 exist to likely play the same role. While their gene orthologs are found in the genomes of *B. mori*, *Tribolium castaneum*, and *T. molitor* (31; data not shown), it is unclear if the corresponding protein products work together to further enhance PPO activation. *T. molitor* and *H. diomphalia* PPAF2 (SPHI) alone largely increased PPO activation (14, 16, 17). While the requirement for the SPHI and SPHII co-presence in the beetles and *A. aegypti* (18) remains unexamined, it is known that *Apis mellifera* genome has one SPHI but no SPHII gene (31). With a single PPO gene, the honeybee PPO activation may represent the simplest reaction among all holometabolous insects. On the other end of the spectrum, the African malaria mosquito, *Anopheles gambiae*, has eleven SPHI, one SPHII, and nine PPO genes in its genome (42). Their roles in melanization, a key immune response against pathogens and parasites, are worth exploring. Lineage-specific expansions of the SPHI and SPHII subfamilies in lepidopteran pests (12) and their functional implications and molecular mechanisms also need to be examined in the future to better understand this defense response.

### 4.2 Cleavage activation of the pro-cSPHs in *D. melanogaster*, *M. sexta*, and *H. diomphalia*

We have not yet identified any endogenous activating protease of pro-cSPH35 or pro-cSPH242 in the fruit fly. *Manduca* PAP3 yielded the fly cSPHs to assist the activation of *Drosophila* PPO1 by MP2x_a_ (**Figures 3**–**5**). Cleavage first occurred at the corresponding site between Cys-3 and Cys-4 of their clip domain in the proSPHI and proSPHII, and then at the junction between the linker and protease-like domain of the SPHI intermediate (4), suggesting that their endogenous activating protease(s) act the same way in *Drosophila*. If proven true, this would be remarkable since Lepidoptera and Diptera separated from each other >250 million years ago and site-1 is located in an exposed loop of the clip domains (48–50). It is likely that cleavage at site-1 leads to the exposure of site-2 in the SPHI to PAP3 for the second cut (**Figure 4A**). Relative roles of these cleavage events in generating an active cofactor (*i.e*., SPHI-II complex) remain unclear and need to be determined biochemically.

The formation of high *M*_r_ complexes is a vital process for pathogen recognition, melanization, and hemolymph coagulation during insect immune responses (51). In this investigation, cleavage-induced oligomerization of cSPH35 and cSPH242 has been clearly demonstrated (**Figures 4C, D**, **S7**). While this agrees well with the positive correlation between native SPHI sizes and cofactor activity in *M. sexta* (12), we noticed a difference between the SPHIIs in these two insects. *Manduca* SPH2 had much lower association states and, therefore, may attach to the large scaffolds of SPHIs (*e.g*., 1b and 101), whereas *Drosophila* cSPH242 and cSPH35 oligomers had similar *M*_r_’s and could further associate with each other (**Figure S7**, lanes 7, 9, and 10) for PPO attachment, PAP cleavage, and PO oligomerization (8). Unlike the two-hexameric-toroidal structure of *H. diomphalia* PPAF2 (48), no such ordered assembly (*e.g*., hexamer) was detected as a prominent form among the *D. melanogaster* SPHI or SPHII complexes (**Figures 4C, D**). Instead, they appeared to grow in size by adding one SPH unit at a time, as suggested in the analysis of association states of cSPH35 and cSPH242 by native gel electrophoresis (**Table 1**). Oligomers of the two cSPHs are further associated with higher *M*_r_ complexes (**Figure S7**, lanes 7, 9, and 10) and their co-presence is required for displaying the maximal PO activity (**Figure 5C**). Consistent with the association, the cofactor isolated from hemolymph of *M. sexta* pharate pupae had an average *M*_r_ of 790 kDa, with a continuous distribution from the void volume of the Sephacryl S500 column (11).

### 4.3 Proteolytic cleavage and PPO activation in various insects

Not all PPO cleavages generate active PO and extra cautions are necessary when a PO activity is assigned to (a) specific cleavage product(s) due to several reasons. First of all, PPO cleavage is essential for *in vivo* activation (46). Cationic surfactants (*e.g*., cetylpyridinium chloride) and organic solvents (*e.g*., 30% ethanol) do activate PPOs *in vitro* (52), but that is not physiologically relevant. Secondly, there is no direct correlation between cleavage extent and PO activity level (**Figure 5**, 46). *Drosophila* MP2x_a_ directly cleaved 50% of the recombinant PPO1 (0.38 μg) but no PO activity was reported (29). On the other hand, since there is a single cleavage in PPO1 probably at the classical activation site between Arg^52^ and Phe^53^, the 73 kDa cleavage product and its assemblies are responsible for the high PO-specific activity (**Figure 5**, lane 13). There are reports for active POs at other sizes, such as 60 kDa PO of *H. diomphalia* (14), 50 kDa PO3 of *A. aegypti* (53), and 60 kDa PO1 of *D. melanogaster* (52). Unfortunately, there is a lack of explicit experimental evidence for these claims. For instance, besides the appearance of the 60 kDa cleavage product, there was a clear intensity increase of the 75 kDa band to account for the boost of PPO activation when *H. diomphalia* PPAF2 was present (14). In the other cases, only low levels of PO activity were detected, probably due to the absence of a fully functional cofactor of SPHI and SPHII. Thirdly, PPO activation occurs only when PPO, active PAP, and its cofactor coexist (46), suggesting that proper association and maintenance of active conformation is crucial for producing PO with high specific activity (**Figure 5**). So far, mechanisms for the generation of highly active PO are unclear but, as more cofactors of PPO activation are prepared *in vitro* from proSPHI and proSPHII, details of the molecular interactions among PPO, PAP, and cofactor are expected to be uncovered in more insects. In *D. melanogaster*, the knockdown of cSPH35 or cSPH242 expression significantly reduced melanization in adults (**Figures 6**, **7**), similar to the phenotype of PPO1 and PPO2 knockouts (20). It is worth testing if cSPH35 and cSPH242 are also required for PPO2 activation and what specific activity of PO is generated under pathological conditions for comparison with the *in vitro* data (260 U/μg).

### 4.4 Conclusions

Proteolytic activation of PPO is required for the survival of *D. melanogaster* during a microbial infection. This process involves PPO, its activating protease, and a high *M*_r_ cofactor (**Figure 8**). In the reaction of MP2 and PPO1 only, the PAP cleaves the substrate likely between Arg^52^ and Phe^53^ to generate active PO1, which seems to quickly lose its activity. The cofactor of cSPH35 and cSPH242 increases the PPO1 cleavage and may maintain PO1 in its active conformation. *M. sexta* PAP3 cleaves pro-cSPH35 at Arg^81^ and then at Lys^128^ to cause cSPH35 association. PAP3 processing of pro-cSPH242 at Arg^88^ also induces oligomerization of cSPH242. An endogenous activating protease, currently unknown, may play the role of PAP3 to generate the cofactor in *Drosophila*. In the presence of the SPHI and SPHII complexes formed *in vitro*, MP2x_a_-treated PPO1 reached an unprecedentedly high PO-specific activity of 260 U/μg. Coupling active, oligomeric PO near the site of injury or infection through the high *M*_r_ cofactor may ensure melanization occurs as a potent, local defense response against pathogens. On the other hand, a rapid activity loss of the diffusing PO could minimize the damage to distant host cells or tissues. These biochemical data are consistent with the *in vivo* test results. The lower PPO1 cleavage, PO activity, hemolymph melanization, and survival rate highlight the importance of clip-domain SPs and SPHs in this antimicrobial defense response. It is further demonstrated that the combined approach, guided by orthologous relationships and expression patterns, is successful in the elucidation of mechanisms for complex physiological processes in the genetic model species.

## Data availability statement

The original contributions presented in the study are publicly available. This data can be found here: https://doi.org/10.6084/m9.figshare.23786949.

## Ethics statement

Ethical review and approval was not required for the study on animals in accordance with the local legislation and institutional requirements.

## Author contributions

QJ designed and did most of the experiments, and she also prepared the draft of the manuscript. HJ guided the work, he provided funds and mainly focused on writing the manuscript. YW purified and provided a lot of important proteins. HY helped to do some important *in vivo* experiments. All authors contributed to the article and approved the submitted version.

## Supplementary material

Supplementary Figure 1 Phylogenetic relationships of the SPHI and SPHII subfamily members in *Drosophila* spp., *Bactrocera oleae*, *Ceratitis capitata*, *Musca domestica*, and *Manduca sexta*. BLASTP searches of GenBank, hit examination, alignment of entire sequences, and construction of the neighbor-joining tree were performed as described before (12). Species names and their abbreviations in parenthesis are *Drosophila albomicans* (Dal), *D. elegans* (Del), *D. erecta* (Der), *D. grimshawi* (Dgr), *D. innubila* (Din), *D. mauritiana* (Dma), *D. melanogastor* (Dme), *D. persimilis* (Dpe), *D. pseudoobscura* (Dps), *D. sechellia* (Dse), *D. suzukii* (Dsu), *D. virilis* (Dvi), *D. willistoni* (Dwi), *D. yakuba* (Dya), *B. oleae* (Bol), *C. capitata* (Cca), *M. domestica* (Mdo), and *M. sexta* (Mse). Suggested protein names consist of an abbreviated species name and a group name of SPHI or SPHII. The *M. sexta* SPHIs (*i.e.*, 1a, 1b, 4, and 101) and SPHII (*i.e*., 2) are in *bold* font, and so are *D. melanogastor* cSPH242 and cSPH35. Duplicated genes in a single species are labeled by “‡”. Percentage bootstrap values greater than 50% are marked near branching points of the tree, with values ≥99 simplified as “*”.

Supplementary Figure 2 Expression profiles of *D. melanogastor* cSPH35 and cSPH242 genes in different tissue samples and developing stages. **(A)** The relative mRNA levels, as represented by FPKM values (31), are shown in bar graphs. The stages labeled “*” roughly correspond to the L, P, and F stages in . The 52 cDNA libraries were constructed from various tissues and stages, which are abbreviated using: W, whole insect; E, embryo; L1–3, 1^st^, 2^nd^, and 3^rd^ instar larval; PS, puff stage; preP, prepupal; P, pupal; A, adult; M, male; F, female; h, hour; D, day; G, gut; PG, posterior midgut; FB, fat body; ID, imaginal discs, SG, salivary glands; T, testes; O, ovaries of adult mated female; AG-AmM, accessory glands of adult mated male; AvF, adult virgin female; MT, Malpighian tubules. **(B)** Abundances of cSPH35 and cSPH242 proteins (44) are shown in the bar graphs as log_2_(LFQ/5×10^6^ + 1) values (mean ± SEM, n = 4). L3c, crawling 3^rd^ instar larvae.

Supplementary Figure 3 Activation of proMP2x_a_ by Factor X_a_ and amidase activity of the MP2x_a_. **(A)** immunoblot. As described in *Section 2.7*, the purified proMP2x_a_ was treated with Factor Xa in 10 μl buffer A. Half of the mixture and controls were subjected to 10% SDS-PAGE followed by immunoblot analysis using hexahistidine antibody. The MP2x_a_ precursor (*) and catalytic domain (*arrowhead*) are labeled. **(B)** Amidase activity. After IEAR*p*NA was added to the remaining samples, substrate hydrolysis was monitored in a kinetic mode on a plate reader. IEARase activities (mean ± SEM, n = 3) are shown in a bar graph. *, *p <*0.05 in Student’s t-test.

Supplementary Figure 4 MP2x_a_ did not significantly activate the cSPH35 and cSPH242 precursors. After proMP2x_a_ (0.44 μg) was preincubated with Factor Xa (0.1 μg) for 1 h at 37 °C in 11 μl buffer A, 1.0 μl of the mixture was added to purified pro-cSPH35 or pro-cSPH242 (200 ng/μl, 1 μl) in 10 μl buffer A. After incubation at 37°C for 1 h, the reaction mixtures and controls were either treated with 1×SDS sample buffer at 95°C for 5 min prior to 10% SDS-PAGE or mixed with the sample buffer lacking SDS and DTT prior to 10% native PAGE. After electrotransfer, immunoblot analysis was performed using 1:1000 diluted antiserum against hexahistidine tag. In panels **(A, B)**, pro-cSPH35 or pro-cSPH242 were marked by “*”. Positions and sizes of the prestained *M*_r_ standards are indicated, with the 75 kDa marker highlighted *red*. In panel **(C)**, the dashed line divides the stacking and separating gels. The bands of MP2x_a_, pro-cSPH35, and pro-cSPH242 were marked by *green*, *blue*, and *red vertical bars*, respectively.

Supplementary Figure 5 *M. sexta* serpin-3 inhibited PAP3 but not *Drosophila* MP2x_a_. **(A)** Active MP2x_a_ (0.44 μg) was first generated as described in **Figure S4**. *M. sexta* serpin-3 (1 μl, 0.3 μg/μl) was then added to the mixture and incubated for 30 min at room temperature. The reaction mixture and controls were treated with 1×SDS sample buffer at 95°C for 5 min prior to 10% SDS-PAGE and immunoblot analysis using 1:1000 diluted antiserum against hexahistidine tag. MP2x_a_ precursor and catalytic domain were marked *asterisk* and *arrowhead*, respectively. **(B)** Active PAP3 (0.1 μg) (*left*) or MP2x_a_ (0.22 μg) (*right*) was incubated with *M. sexta* serpin-3 (0.3 μg) at room temperature for 30 minutes. As described in *Section 2.7*, IEARase activities (mean ± SEM, n = 3) were measured and plotted as bar graphs. Pairwise comparisons were performed between control and treated samples by Student’s t-test. *, *p <*0.05; ns, not significant (*p >*0.05).

Supplementary Figure 6 *D. melanogastor* PPO1 cleavage by MP2x_a_ in the presence of pro-cSPH35 and pro-cSPH242. ProMP2x_a_ (0.44 μg) was first activated by Factor Xa (0.1 μg) in 2 μl buffer A at 37°C for 1 h. The purified PPO1 (1 μl, 0.3 μg/μl) from *E. coli* was incubated on ice for 1 h with the active MP2x_a_ (0.2 μg) in the presence of pro-cSPH35 and pro-cSPH242 (0.2 μg each) in 10 μl buffer A on ice for 1h. Half of the mixture (lane 11) and controls (lanes 1–10) were subjected to 7% SDS-PAGE followed by immunoblot analysis using specific antibodies to PPO1 (26). Missing components (s) in the controls were replaced by the same volume of buffer A.

Supplementary Figure 7 Mobility changes accompanying the cSPH cleavage and association analyzed by native PAGE. **(A)** An example of the stained gels. The purified pro-cSPH35 and pro-cSPH242 (200 ng/μl, 5 μl for each or 5 + 5 μl for both) were incubated with PAP3 (40 ng/μl, 5 μl) in 50 μl buffer A for 1 h at 37°C. One-fifth of the three mixtures and two controls of pro-cSPH35 and pro-cSPH242 alone were separated along with *M*_r_ standards by 4.5%, 7%, 10%, 12%, and 15% native discontinuous PAGE, followed by Coomassie staining. The standard proteins (29 kDa carbonic anhydrase, 67 kDa bovine serum albumin, 200 kDa β-amylase, 443 kDa apoferritin, and 669 kDa thyroglobulin in lanes 1–5), proSPHs (in lanes 6 and 8), and new bands (in lanes 7, 9, and 10) are marked with “●”, “*” and *arrowheads*, respectively. **(B)** Native molecular mass (*M*_r_) standard curve. **(C)** Parameters of the pro-cSPH and cSPH bands. For a specific form or association state, the slope (*K*_r_) of the log_10_R_m_ (relative mobility) vs. %T (total monomer concentration) curve from its corresponding bands on the gels was used to derive its unknown mass using the standard curve on Ferguson plot (37). Estimated *M*_r_’s of the complexes of cleaved cSPH35, and cSPH242, and both are in *bold* font.

Supplementary Figure 8 Secondary ion mass spectra of the characteristic peptides from the PAP3-treated cSPHs after V8 **(A, B)** or chymotrypsin **(C)** proteolytic digestion. Based on the peptide sequences and their locations in the proSPH sequences, the cleavage sites of *M. sexta* PAP3 were deduced and shown in.

## References

[1] Nappi AJ Christensen BM. Melanogenesis and associated cytotoxic reactions: applications to insect innate immunity. Insect Biochem Mol Biol (2005) 35:443–59. doi: 10.1016/j.ibmb.2005.01.014 15804578

[2] Jiang H Vilcinskas A Kanost MR. Immunity in lepidopteran insects. Adv Exp Med Biol (2010) 708:181–204. doi: 10.1007/978-1-4419-8059-5_10 21528699 PMC9284565

[3] Park JW Kim CH Rui J Park KH Ryu KH Chai JH. Beetle immunity. In: Söderhäll K, editor. Invertebrate Immunity. Adv Exp Med Biol, New York, NY:Springer. vol. 708 (2010). p. 163–80. 10.1007/978-1-4419-8059-5_9 21528698

[4] Kanost MR Jiang H. Clip-domain serine proteases as immune factors in insect hemolymph. Curr Opin Insect Sci (2015) 11:47–55. doi: 10.1016/j.cois.2015.09.003 26688791 PMC4680995

[5] Marieshwari BN Bhuvaragavan S Sruthi K Mullainadhan P Janarthanan S. Insect phenoloxidase and its diverse roles: melanogenesis and beyond. J Comp Physiol B (2023) 193:1–23. doi: 10.1007/s00360-022-01468-z 36472653

[6] Satoh D Horii A Ochiai M Ashida M. Prophenoloxidase activating enzyme of the silkworm, *Bombyx mori*: purification, characterization, and cDNA cloning. J Biol Chem (1999) 274:7441–53. doi: 10.1074/jbc.274.11.7441 10066809

[7] Jiang H Wang Y Kanost MR. Pro-phenoloxidase activating proteinase from an insect, Manduca sexta: a bacteria-inducible protein similar to Drosophila easter. Proc Natl Acad Sci USA (1998) 95:12220–5. 10.1073/pnas.95.21.12220 PMC22812 9770467

[8] Jiang H Wang Y Yu XQ Kanost MR. Prophenoloxidase-activating proteinase-2 from hemolymph of *Manduca sexta*: a bacteria-inducible serine proteinase containing two clip domains. J Biol Chem (2003) 278:3552–61. doi: 10.1074/jbc.M205743200 12456683

[9] Jiang H Wang Y Yu XQ Zhu Y Kanost MR. Prophenoloxidase-activating proteinase-3 from *Manduca sexta* hemolymph: a clip-domain serine proteinase regulated by serpin-1J and serine proteinase homologs. Insect Biochem Mol Biol (2003) 33:1049–60. doi: 10.1016/S0965-1748(03)00123-1 14505699

[10] Yu XQ Jiang H Wang Y Kanost MR. Nonproteolytic serine protease homologs are involved in prophenoloxidase activation in the tobacco hornworm, *Manduca sexta* . Insect Biochem Mol Biol (2003) 33:197–208. doi: 10.1016/S0965-1748(02)00191-1 12535678

[11] Wang Y Jiang H. Prophenoloxidase (proPO) activation in *Manduca sexta*: an analysis of molecular interactions among proPO, proPO-activating proteiase-3, and a cofactor. Insect Biochem Mol Biol (2004) 34:731–42. doi: 10.1016/j.ibmb.2004.03.008 15262278

[12] Jin Q Wang Y Hartson SD Jiang H. Cleavage activation and functional comparison of *Manduca sexta* serine protease homologs SPH1a, SPH1b, SPH4, and SPH101 in conjunction with SPH2. Insect Biochem Mol Biol (2022) 144:103762. doi: 10.1016/j.ibmb.2022.103762 35395380 PMC9328667

[13] Wang Q Yin M Yuan C Liu X Hu Z Zou Z. Identification of a conserved prophenoloxidase activation pathway in cotton bollworm *Helicoverpa armigera* . Front Immunol (2020) 11:785. doi: 10.3389/fimmu.2020.00785 32431706 PMC7215089

[14] Lee SY Kwon TH Hyun JH Choi JS Kawabata SI Iwanaga S. *In vitro* activation of prophenoloxidase by two kinds of prophenoloxidase activating factors isolated from hemolymph of coleopteran, *Holotrichia diomphalia* larvae. Eur J Biochem (1998) 254:50–7. doi: 10.1046/j.1432-1327.1998.2540050.x 9652393

[15] Kwon TH Kim MS Choi HW Joo CH Cho MY Lee BL. A masquerade-like serine proteinase homologue is necessary for phenoloxidase activity in the coleopteran insect, *Holotrichia diomphalia* larvae. Eur J Biochem (2000) 267:6188–96. doi: 10.1046/j.1432-1327.2000.01695.x 11012672

[16] Lee KY Zhang R Kim MS Park JW Park HY Kawabata S. A zymogen form of masquerade-like serine proteinase homologue is cleaved during pro-phenoloxidase activation by Ca^2+^ in coleopteran and *Tenebrio molitor* larvae. Eur J Biochem (2002) 269:4375–83. doi: 10.1046/j.1432-1033.2002.03155.x 12199717

[17] Kan H Kim CH Kwon HM Park JW Roh KB Lee H. Molecular control of phenoloxidase-induced melanin synthesis in an insect. J Biol Chem (2008) 283:25316–23. doi: 10.1074/jbc.M804364200 18628205

[18] Ji Y Lu T Zou Z Wang Y. *Aedes aEgypti* CLIPB9 activates prophenoloxidase-3 in the presence of CLIPA14 after fungal infection. Front Immunol (2022) 13:927322. doi: 10.3389/fimmu.2022.927322 35967454 PMC9365933

[19] Binggeli O Neyen C Poidevin M Lemaitre B. Prophenoloxidase activation is required for survival to microbial infections in *Drosophila* . PLoS Pathog (2014) 10:e1004067. doi: 10.1371/journal.ppat.1004067 24788090 PMC4006879

[20] Dudzic JP Kondo S Ueda R Bergman CM Lemaitre B. *Drosophila* innate immunity: regional and functional specialization of prophenoloxidases. BMC Biol (2015) 13:81. doi: 10.1186/s12915-015-0193-6 26437768 PMC4595066

[21] Nam HJ Jang IH Asano T Lee WJ. Involvement of prophenoloxidase 3 in lamellocyte-mediated spontaneous melanization in *Drosophila* . Mol Cells (2008) 26:606–10. 18852525

[22] Castillejo-López C Häcker U. The serine protease Sp7 is expressed in blood cells and regulates the melanization reaction in *Drosophila* . Biochem Biophys Res Commun (2005) 338:1075–82. doi: 10.1016/j.bbrc.2005.10.042 16256951

[23] Leclerc V Pelte N El Chamy L Martinelli C Ligoxygakis P Hoffmann JA. Prophenoloxidase activation is not required for survival to microbial infections in *Drosophila* . EMBO Rep (2006) 7:231–5. doi: 10.1038/sj.embor.7400592 PMC1369246 16322759

[24] Tang H Kambris Z Lemaitre B Hashimoto C. Two proteases defining a melanization cascade in the immune system of *Drosophila* . J Biol Chem (2006) 281:28097–104. doi: 10.1074/jbc.M601642200 16861233

[25] Ayres JS Schneider DS. A signaling protease required for melanization in *Drosophila* affects resistance and tolerance of infections. PLoS Biol (2008) 6:2764–73. doi: 10.1371/journal.pbio.0060305 PMC2596860 19071960

[26] Nam HJ Jang IH You H Lee KA Lee WJ. Genetic evidence of a redox-dependent systemic wound response via Hayan protease-phenoloxidase system in Drosophila. EMBO J (2012) 31:1253–65. doi: 10.1038/emboj.2011.476 PMC3297987 22227521

[27] Zhao P Li J Wang Y Jiang H. Broad-spectrum antimicrobial activity of the reactive compounds generated in *vitro* by *Manduca sexta* phenoloxidase. Insect Biochem Mol Biol (2007) 37:952–9. doi: 10.1016/j.ibmb.2007.05.001 PMC2047599 17681234

[28] Zhao P Lu Z Strand MR Jiang H. Antiviral, anti-parasitic, and cytotoxic effects of 5, 6-dihydroxyindole (DHI), a reactive compound generated by phenoloxidase during insect immune response. Insect Biochem Mol Biol (2011) 41:645–52. doi: 10.1016/j.ibmb.2011.04.006 PMC3129360 21554953

[29] An C Zhang M Chu Y Zhao Z. Serine protease MP2 activates prophenoloxidase in the melanization immune response of *Drosophila melanogaster* . PLoS One (2013) 8:e79533. doi: 10.1371/journal.pone.0079533 24260243 PMC3829845

[30] Li X Ma M Liu F Chen Y Lu A Ling QZ. Properties of *Drosophila melanogaster* prophenoloxidases expressed in *Escherichia coli* . Dev Comp Immunol (2012) 36:648–56. doi: 10.1016/j.dci.2011.11.005 22120533

[31] Cao X Jiang H. Building a platform for predicting functions of serine protease-related proteins in *Drosophila melanogaster* and other insects. Insect Biochem Mol Biol (2018) 103:53–69. doi: 10.1016/j.ibmb.2018.10.006 30367934 PMC6358214

[32] Wang Y Lu Z Jiang H. *Manduca sexta* proprophenoloxidase activating proteinase-3 (PAP3) stimulates melanization by activating proPAP3, proSPHs, and proPOs. Insect Biochem Mol Biol (2014) 50:82–91. doi: 10.1016/j.ibmb.2014.04.005 24768974 PMC4064829

[33] Brand AH Perrimon N. Targeted gene expression as a means of altering cell fates and generating dominant phenotypes. Development (1993) 118:401–15. doi: 10.1242/dev.118.2.401 8223268

[34] Kapila R Poddar S Meena N Prasad NG. Investment in adult reproductive tissues is affected by larval growth conditions but not by evolution under poor larval growth conditions in Drosophila melanogaster. Curr Res Insect Sci (2021) 2:100027. doi: 10.1016/j.cris.2021.100027 36003263 PMC9387493

[35] Lu Z Jiang H. Expression of *Manduca sexta* serine proteinase homolog precursors in insect cells and their proteolytic activation. Insect Biochem Mol Biol (2008) 38:89–98. doi: 10.1016/j.ibmb.2007.09.011 18070668 PMC2199269

[36] Sumathipala N Jiang H. Involvement of *Manduca sexta* peptidoglycan recognition protein-1 in the recognition of bacteria and activation of prophenoloxidase system. Insect Biochem Mol Biol (2010) 40:487–95. doi: 10.1016/j.ibmb.2010.04.008 PMC2931796 20416376

[37] Gallagher SR. One-dimensional electrophoresis using nondenaturing conditions. In: Coligan JE Dunn BM Ploegh HL Speicher DW Wingfield PT, editors. Current Protocols in Protein Science. Hoboken, New Jersey:Wiley (1995). p. 10.3.5–10.3.11.

[38] Zhang S Cao X He H Hartson S Jiang H. Semi-quantitative analysis of changes in the plasma peptidome of *Manduca sexta* larvae and their correlation with the transcriptome variations upon immune challenge. Insect Biochem Mol Biol (2014) 47:46–54. doi: 10.1016/j.ibmb.2014.02.002 24565606 PMC3992937

[39] Cao X Wang Y Rogers J Hartson S Kanost MK Jiang H. Changes in composition and levels of hemolymph proteins during metamorphosis of *Manduca sexta* . Insect Biochem Mol Biol (2020) 127:103489. doi: 10.1016/j.ibmb.2020.103489 33096211 PMC7704632

[40] Chapman JR Dowell MA Chan R Unckless RL. The genetic basis of natural variation in *Drosophila melanogaster* immune defense against *Enterococcus faecalis* . Genes (Basel) (2020) 11:234. doi: 10.3390/genes11020234 32098395 PMC7074548

[41] Troha K Im JH Revah J Lazzaro BP Buchon N. Comparative transcriptomics reveals CrebA as a novel regulator of infection tolerance in D. melanogaster. PLoS Pathog (2018) 14:e1006847. doi: 10.1371/journal.ppat.1006847 29394281 PMC5812652

[42] Cao X Gulati M Jiang H. Serine protease-related proteins in the malaria mosquito, *Anopheles Gambiae* . Insect Biochem Mol Biol (2017) 88:48–62. doi: 10.1016/j.ibmb.2017.07.008 28780069 PMC5586530

[43] He Y Cao X Zhang S Rogers J Hartson S Jiang H. Changes in the plasma proteome of Manduca sexta larvae in relation to the transcriptome variations after an immune challenge: evidence for high molecular weight immune complex formation. Mol Cell Proteomics (2016) 15:1176–87. 10.1074/mcp.M115.054296 PMC4824848 26811355

[44] Casas-Vila N Bluhm A Sayols S Dinges N Dejung M Altenhein T. The developmental proteome of *Drosophila melanogaster* . Genome Res (2017) 27:1273–85. doi: 10.1101/gr.213694.116 PMC5495078 28381612

[45] Zhu Y Wang Y Gorman M Jiang H Kanost MR. *Manduca sexta* serpin-3 regulates prophenoloxidase activation in response to infection by inhibiting prophenoloxidase activating proteinases. J Biol Chem (2003) 47:46556–64. doi: 10.1074/jbc.M309682200 12966082

[46] Gupta S Wang Y Jiang H. *Manduca sexta* prophenoloxidase (proPO) activation requires proPO-activating proteinase (PAP) and serine proteinase homologs (SPHs) simultaneously. Insect Biochem Mol Biol (2005) 35:241–8. doi: 10.1016/j.ibmb.2004.12.003 PMC2042486 15705503

[47] Zakhia R Osta MA. CLIPA7 exhibits pleiotropic roles in the *Anopheles Gambiae* immune response. J Innate Immun (2022) 24:1–16. doi: 10.1159/000526486 PMC10643895 36423593

[48] Piao S Song YL Kim JH Park SY Park JW Lee BL. Crystal structure of a clip-domain serine protease and functional roles of the clip domains. EMBO J (2005) 24:4404–14. doi: 10.1038/sj.emboj.7600891 PMC1356332 16362048

[49] Huang R Lu Z Dai H Velde DV Prakash O Jiang H. The solution structure of the clip domains from *Manduca sexta* prophenoloxidase activating proteinase-2. Biochemistry (2007) 46:11431–9. doi: 10.1021/bi7010724 17880110

[50] Cao X He Y Hu Y Zhang X Wang Y Zou Z. Sequence conservation, phylogenetic relationships, and expression profiles of nondigestive serine proteases and serine protease homologs in *Manduca sexta* . Insect Biochem Mol Biol (2015) 62:51–63. doi: 10.1016/j.ibmb.2014.10.006 25530503 PMC4474797

[51] Clark KD. Insect hemolymph immune complexes. Subcell Biochem (2020) 94:123–61. doi: 10.1007/978-3-030-41769-7_5 32189298

[52] Lu A Li X Hillyer JF Beerntsen BT Söderhäll K Ling E. Recombinant *Drosophila* prophenoloxidase 1 is sequentially cleaved by α-chymotrypsin during in *vitro* activation. Biochimie (2014) 102:154–65. doi: 10.1016/j.biochi.2014.03.007 24657220

[53] Wang Y Jiang H Cheng Y An C Chu Y Raikhel AS. Activation of *Aedes aEgypti* prophenoloxidase-3 and its role in the immune response against entomopathogenic fungi. Insect Mol Biol (2017) 26:552–63. doi: 10.1111/imb.12318 PMC5582978 28556276

