# Supplementary figures and images for: Two clip-domain serine protease homologs, cSPH35 and cSPH242, act as a cofactor for prophenoloxidase-1 activation in Drosophila melanogaster
Source: Front Immunol. 2023 Sep 15;14:1244792. doi: 10.3389/fimmu.2023.1244792 (PMC10540698; doi:10.3389/fimmu.2023.1244792)

Fig. S1

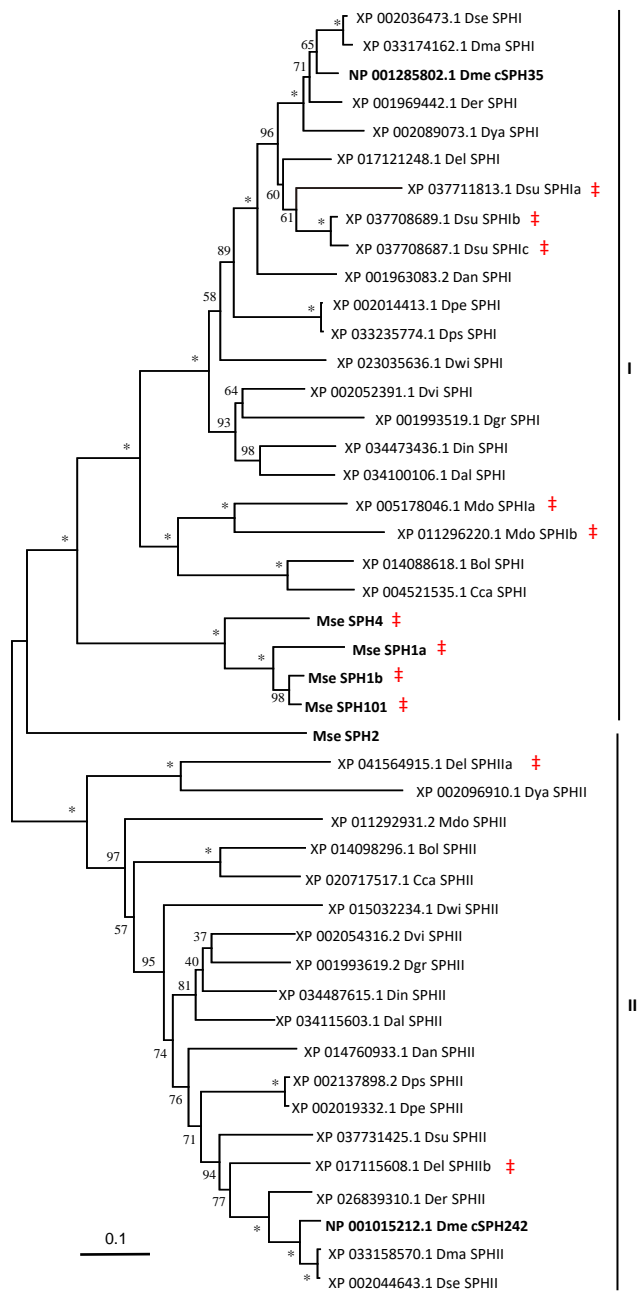

Fig. S2

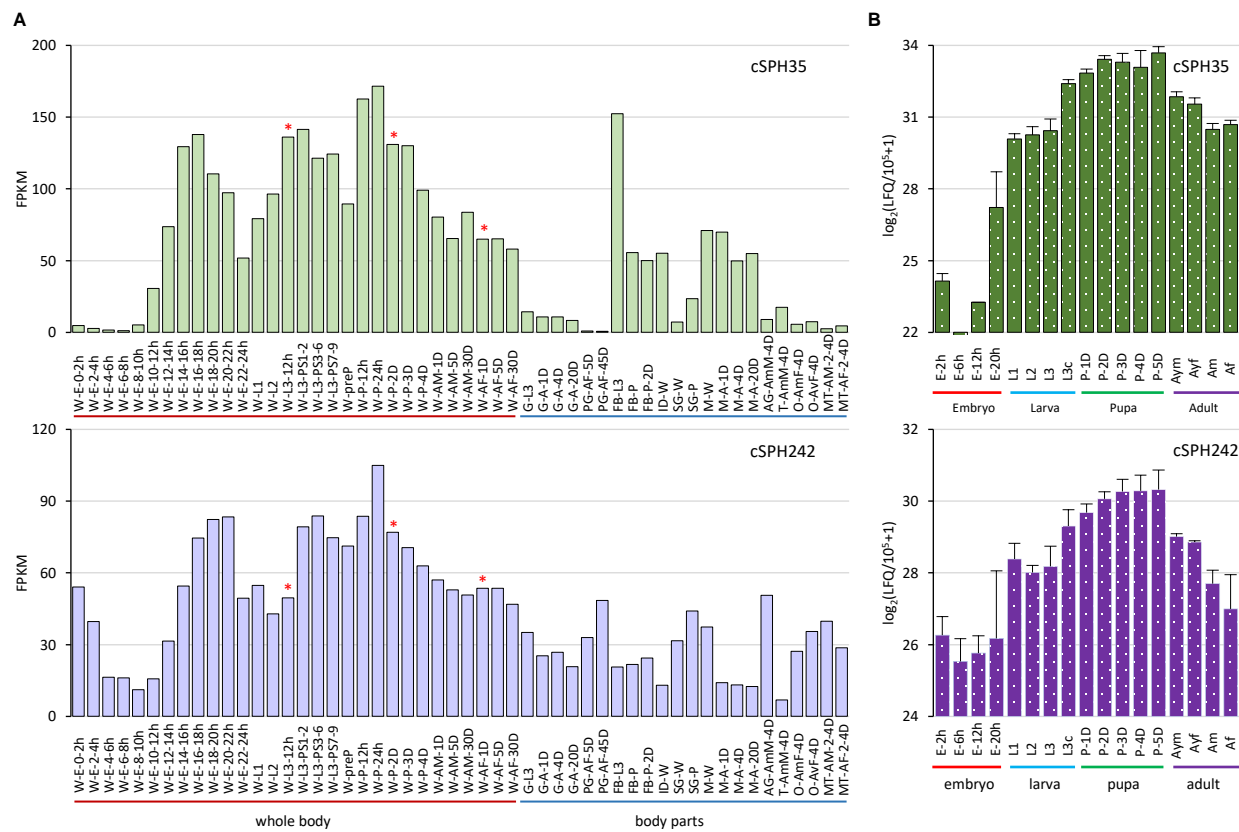

Fig. S3

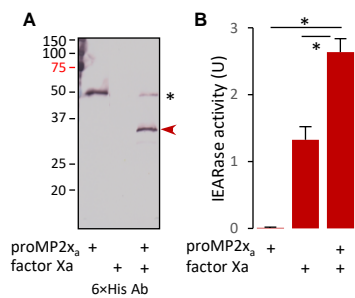

Fig. S4

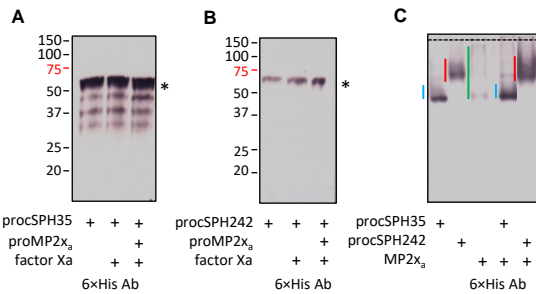

Fig. S5

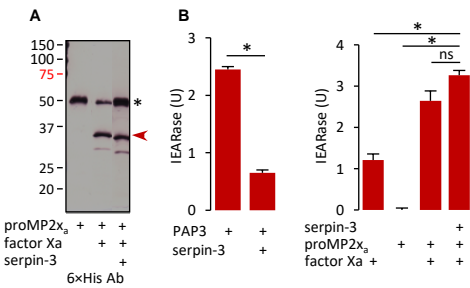

Fig. S6

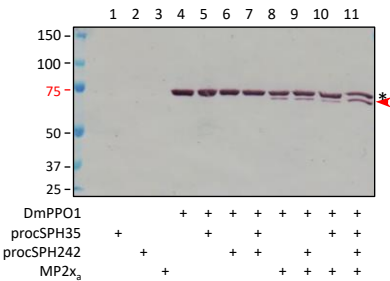

Fig. S7

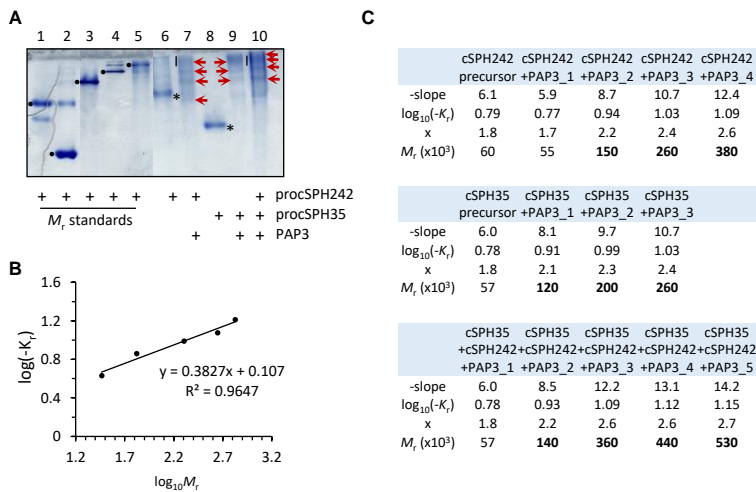

Fig. S8

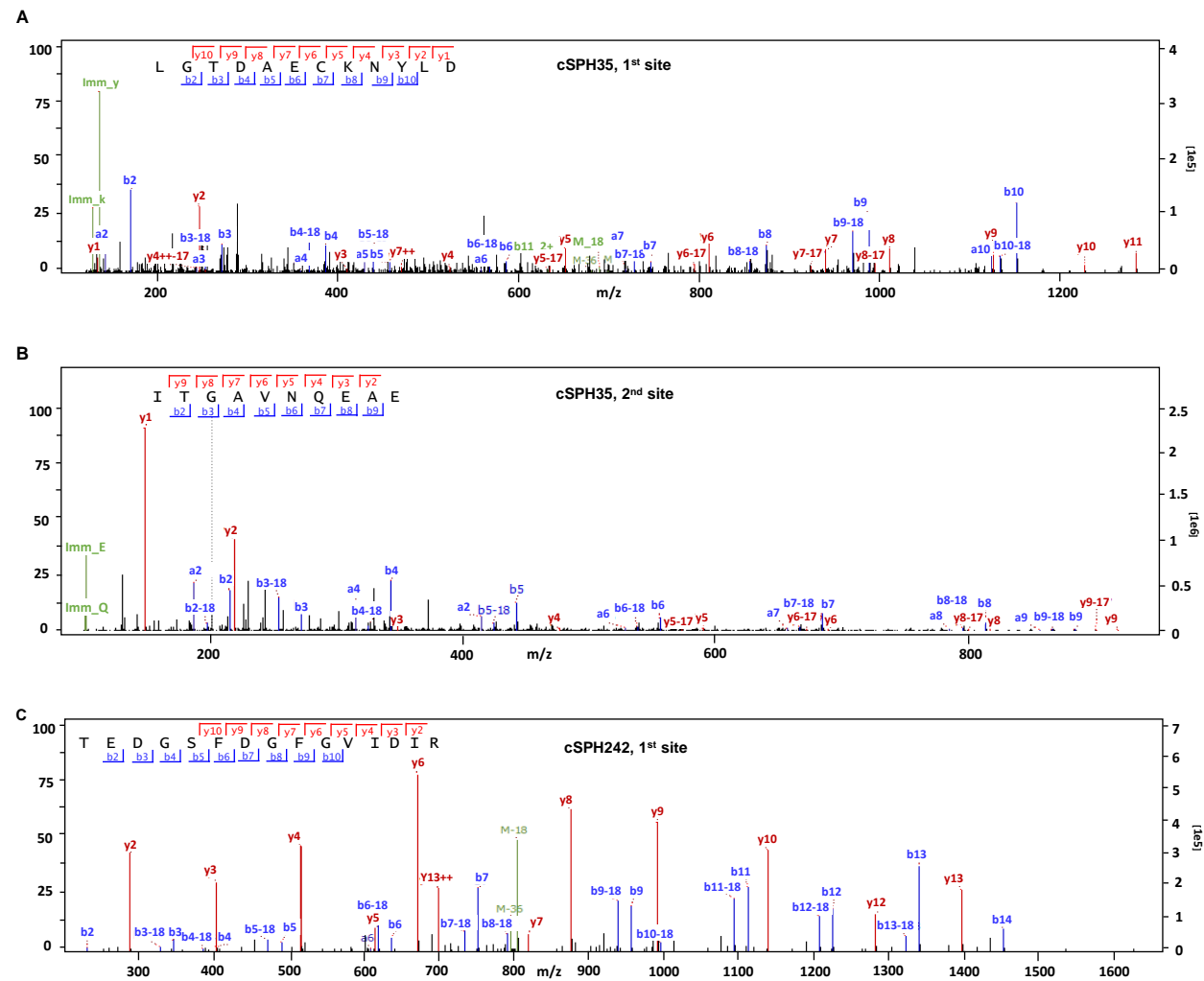

Supplement: Supplementary Figure 1 — Phylogenetic relationships of the SPHI and SPHII subfamily members in Drosophila spp., Bactrocera oleae, Ceratitis capitata, Musca domestica, and Manduca sexta. BLASTP searches of GenBank, hit examination, alignment of entire sequences, and construction of the neighbor-joining tree were performed as described before (12). Species names and their abbreviations in parenthesis are Drosophila albomicans (Dal), D. elegans (Del), D. erecta (Der), D. grimshawi (Dgr), D. innubila (Din), D. mauritiana (Dma), D. melanogastor (Dme), D. persimilis (Dpe), D. pseudoobscura (Dps), D. sechellia (Dse), D. suzukii (Dsu), D. virilis (Dvi), D. willistoni (Dwi), D. yakuba (Dya), B. oleae (Bol), C. capitata (Cca), M. domestica (Mdo), and M. sexta (Mse). Suggested protein names consist of an abbreviated species name and a group name of SPHI or SPHII. The M. sexta SPHIs (i.e., 1a, 1b, 4, and 101) and SPHII (i.e., 2) are in bold font, and so are D. melanogastor cSPH242 and cSPH35. Duplicated genes in a single species are labeled by “‡”. Percentage bootstrap values greater than 50% are marked near branching points of the tree, with values ≥99 simplified as “*”. [file Presentation_1.pdf]
